# The outcomes of total hip replacement in osteonecrosis versus osteoarthritis: a systematic review and meta-analysis

**DOI:** 10.1007/s00264-023-05761-6

**Published:** 2023-03-11

**Authors:** Loay A. Salman, Ashraf T. Hantouly, Harman Khatkar, Abdallah Al-Ani, Abedallah Abudalou, Mohammed Al-Juboori, Ghalib Ahmed

**Affiliations:** 1 Orthopedics Department, Hamad General Hospital, Hamad Medical Corporation, PO Box 3050, Doha, Qatar; 2 Nuffield Department of Orthopaedics, Rheumatology and Musculoskeletal Sciences, Botnar Research Centre, University of Oxford, Windmill Road, Oxford, OX3 7LD UK; 3 Royal London Hospital, Whitechapel, London, UK; 4 Office of Scientific Affairs and Research, King Hussein Cancer Center, Amman, Jordan

**Keywords:** Avascular necrosis of femoral head, Osteoarthritis, Total hip arthroplasty, Revision, Functional outcome

## Abstract

**Purpose:**

This systematic review and meta-analysis aimed to compare the outcomes of THA in patients with osteonecrosis (ON) and those with osteoarthritis (OA).

**Methods:**

Four databases were searched from inception till December 2022 for original studies that compared the outcomes of THA in ON and OA. The primary outcome was the revision rate; the secondary outcomes were dislocation and Harris hip score. This review was conducted in line with PRISMA guidelines, and the risk of bias was assessed using the Newcastle–Ottawa scale.

**Results:**

A total of 14 observational studies with 2,111,102 hips were included, with a mean age of 50.83 ± 9.32 and 55.51 ± 8.95 for ON and OA groups, respectively. The average follow-up was 7.25 ± 4.6 years. There was a statistically significant difference in revision rate between ON and OA patients in favour of OA (OR: 1.576; 95%CI: 1.24–2.00; *p*-value: 0.0015). However, dislocation rate (OR: 1.5004; 95%CI: 0.92–2.43; *p*-value: 0.0916) and Haris hip score (HHS) (SMD: − 0.0486; 95%CI: − 0.35–0.25; *p*-value: 0.6987) were comparable across both groups. Further sub-analysis adjusting for registry data also showed similar results between both groups.

**Conclusion:**

A higher revision rate, periprosthetic fracture and periprosthetic joint infection following total hip arthroplasty were associated with osteonecrosis of the femoral head compared with osteoarthritis. However, both groups had similar dislocation rates and functional outcome measures. This finding should be applied in context due to potential confounding factors, including patient’s age and activity level.

**Supplementary Information:**

The online version contains supplementary material available at 10.1007/s00264-023-05761-6.

## Introduction

Total hip arthroplasty (THA) has revolutionized the treatment of hip pathologies. It is considered one of the most successful and cost-effective surgical treatments for advanced hip disease [1]. Driven by the increased life expectancy and level of activity of the older population, the frequency of THA has been growing substantially. Nowadays, there are more than 300,000 annual total hip replacements in the USA alone, and this number is projected to double by 2030 [2, 3].

Different underlying aetiologies alter hip biomechanics differently, and thus, the outcomes of THA might vary according to the underlying pathology. Hip primary osteoarthritis (OA) and osteonecrosis of the femoral head (ON) are two distinct pathologies that comprise the main indications for THA [4]. ON of the femoral head is responsible for up to 18% of all THA [5]. The outcomes of THA in ON remain controversial, as it has been reported that these patients, who are usually younger and more active, have higher rates of complications and revision surgeries [6].

Therefore, the purpose of this study was to compare the clinical and functional outcomes of THA in patients with ON to those with hip OA. We hypothesized that there is no significant difference between both groups in terms of revision, functional outcomes, and complication rate.

## Materials and methods

This systematic review was conducted in line with the Preferred Reporting Items for Systematic Reviews and Meta-Analyses (PRISMA) guidelines [7]. A protocol registration was sought in advance on the International Prospective Register of Systematic Reviews (PROSPERO) with the registration number: CRD42022374456.

### Search strategy: outcomes of interest

PubMed/Medline, Ovid, Google Scholar, and Cochrane library databases were searched from inception until December 2022 with the following keywords and their derivatives: total hip replacement OR total hip arthroplasty, AND avascular necrosis AND osteoarthritis AND outcomes. Two authors independently screened the search results based on the title and/or abstract. Conflicts were resolved via a discrepancy meeting with a third, more senior author. A full-text review of articles that met the eligibility criteria was performed, and references of included articles were manually sought to ensure all relevant studies were included.

Revision rate was the primary outcome and is defined as “Any operation performed to add, remove, or modify one or more components of a joint replacement” [8]. The Number of dislocations and validated functional outcome measures, using the HHS [9, 10], were used as secondary outcomes of interest.

### Eligibility criteria

Inclusion criteria:

1. All original comparative, RCTs, and observational studies reporting THR indicated in ON or primary OA
2. Studies with a minimum follow-up period of 90 days
3. All types of THR prosthesis designs

Exclusion criteria:

1. Studies with different indications for THR other than OA or ON
2. Noncomparative or not reporting outcomes or failures by subgroups (i.e., ON vs OA)
3. Review articles, cross-sectional, case series, and reports
4. Preclinical studies
5. Studies with incomplete or unextractable data
6. Studies published in languages other than English

### Data extraction and items

Two independent reviewers used a predesigned data collection sheet in Microsoft Excel to extract data. The extracted demographic data included the first authors’ surnames, study year, design, and location; the mean age of patients, number of participants and hip, age, and type of THR (cementless vs cemented, type of prosthesis, and bearings); follow-up period, number of revisions, complications, functional and radiographic outcomes, statistical tests, and conclusion.

**Fig. 1:**
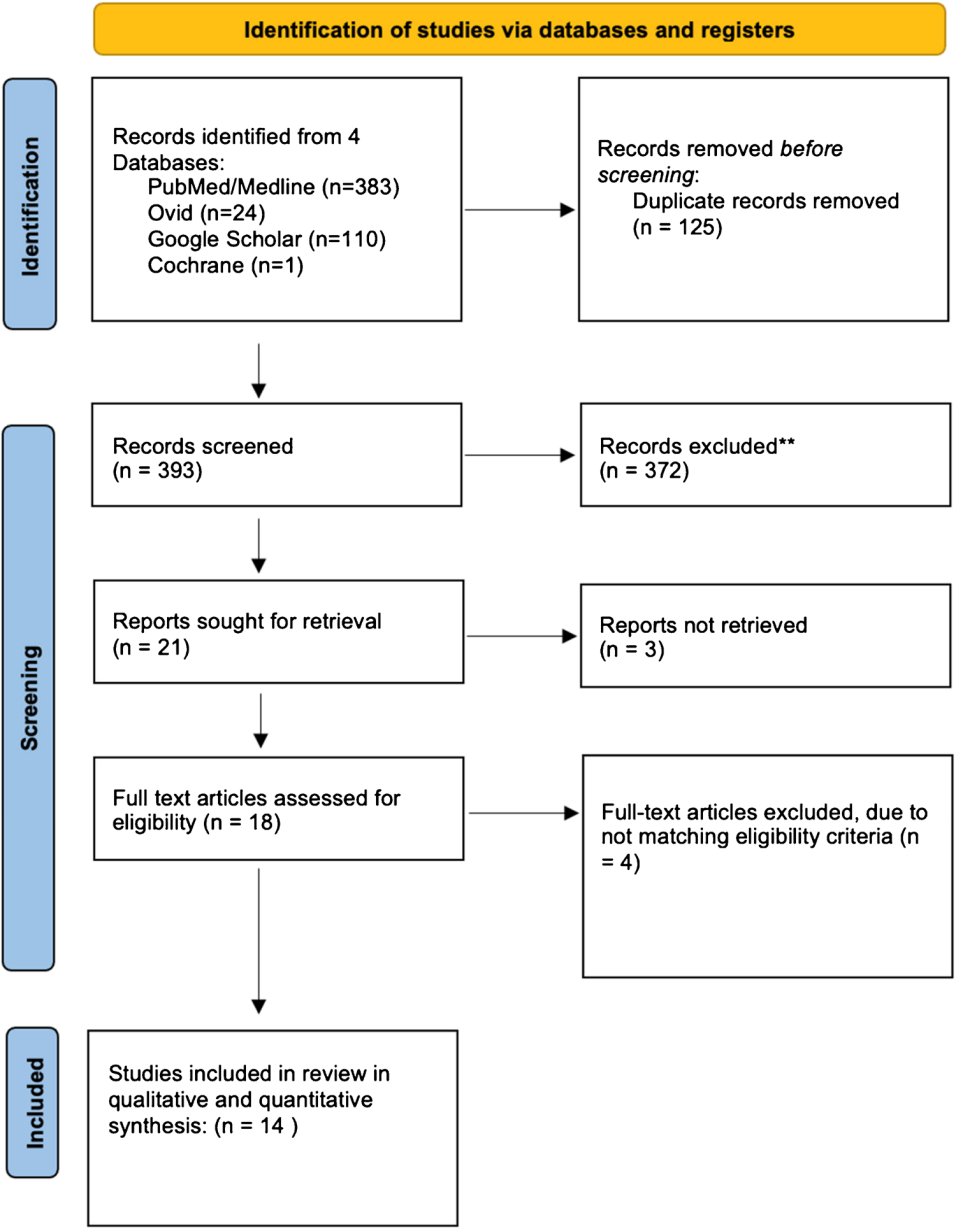
PRISMA flow diagram of record identification, screening and selection in meta-analysis

Rayyan AI website was used to manage the literature search results [11]. Searching the databases yielded 518 articles, and after removing 125 duplicates, 393 records were screened by title and abstracts, of which 372 were excluded. A total of 18 papers were eligible for a full-text review. As a result, 14 studies met the eligibility criteria and were included in the qualitative and quantitative synthesis. The PRISMA flowchart is displayed in Fig. 1.

### Qualitative assessment (risk of bias)

Two of the authors evaluated the methodological quality of the included studies using the Newcastle–Ottawa tool, which is composed of three key domains: patient selection, comparability, and outcomes [12, 13]. The overall risk of bias is then judged as “good,” “fair,” or “poor” quality as per Agency for Healthcare Research and Quality (AHRQ) standards. Data was assessed by at least two authors, and if any disagreement arises, it was resolved by a discussion with a third senior author.

### Quantitative analysis (meta-analysis)

A meta-analysis of eligible studies using R (version 4.0.2, R Core Team, Vienna, Austria, 2020) using the meta package (i.e., forest_meta, metacont, metabin, and metabias functions) was conducted. Odds ratios (OR) and their associated 95% confidence intervals were expressed for dichotomous variables (e.g., number of revisions). For continuous variables (e.g., HHP score), standardized mean differences (SMD) and their associated standardized errors and deviation values were calculated for all eligible studies. Studies that have only provided median values (± range) or isolated mean values, their standard deviation was imputated per the guidelines of Cochrane (refer to Chapter 7.7.3.3) and the methods delineated by Shi et al. (2020), Luo et al. (2018), and Wan et al. (2014). Heterogeneity among effect sizes was evaluated using the *I*-squared statistic. Definitions for heterogeneity were adapted from the Cochrane handbook (> 25% mild, 25–50% moderate, and > 50% severe). Both a funnel plot and Egger’s test of asymmetry were utilized to assess publication bias.

## Results

### Studies characteristics

A total of 14 observational studies (2,111,102 hips) were included in this meta-analysis, with a mean patient age of 50.83 +  − 9.32 and 55.51 +  − 8.95 for AVN and OA groups, respectively. Among these, 12 studies were used to compare the revision rate between patients with ON and OA. While 11 studies assessed the dislocation outcome across both groups, six studies generated a meta-analysis of HHS functional outcome. Twelve studies were retrospective, and two were prospective cohorts. The characteristics of the included studies are summarized in Table 1.

**Table 1:** A summary of baseline study characteristics

Study	Design, LoE	Country	Data	Age (ON/OA)	Gender% (M:F)	#Participants (AVN/OA)	#Hips	THA type	FU (Years)
1997, Xenakis [18]	Retrospective, 3a	Greece	Primary	(51.4, 54.7)	23%:78%	58 (29/29)	74	Cementless	7.6
1999, Ortiguera [31]	Retrospective, 3a	US	Primary	58	37%:63%	158 (79/79)	188	Cemented	17.8
2006, Mont [4]	Retrospective, 3a	US	Primary	(41, 40)	74%:26%	81 (41/40)	104	Cementless	3
2008, Dastane [32]	Retrospective, 3a	US	Primary	(44.7, 51.6)	73%:27%	107 (27/80)	112	Cemntless, cemented	5.5
2009, Radl [33]	Retrospective, 3a	Austria	Primary	(51, 63)	46.3%:53.7%	80 (31/48)	NR	Cementless	6.1
2014, Bergh [16]	Retrospective, 3a	Norway, Denmark, and Sweden	Registry data (NARA)	(65, 69)	41.5%:58.5%	NR	427,806 (11,589/416,217)	Hybrid, cementless, cemented	6.3
2016, Ancelin [21]	Case–Control, 3a	France	Primary	47.8	Male/female ratio, 3.68 (AVN) vs. 1.16 (OA)	282 (149/133)	282	Cementless	11.4
2016, Liu [34]	Retrospective, 3a	Taiwan	Primary	47.1	61.7%:38.3%	402 (216/55)	NR	Cementless, cemented	10
2017, Singh [35]	Prospective, 2b	USA	Registry data (KP TJRR)	66	42.5%:57.5%	47,523 (2271/45,252)		NR	3.2
2018, Osawa [6]	Case–Control, 3a	Japan	Primary	(51.4, 52.2)	40 M, 38F	156 (78/78)	172	Cementless	10
2019, Hart [36]	Retrospective, 3a	US	Primary	59	53%:47%	840	922 (461/461)	Hybrid, cementless	10
2020, Kumar [37]	Retrospective, 3a	India	Primary	43.22	3.2:1	99 (38/15)	118	Cementless, cemented	1.6
2021, Sax [4]	Retrospective, 3a	USA	Registry data (NRD)	(54, 66)	OA: 43.7% M—56.3% F, ON: 57.3% M—42.7% F	1,633,025 (55,034/1,577,991)	NR	NR	NR
2022, Moharrami [38]	Retrospective, 3a	Iran	Primary	(32, 59.6)	ON: 15.9% M—84.1% F, OA: 65.2% M—34.8% F	243 (81/162)	294	Cementless	9

*LoE*, level of evidence; *FU (Y)*, follow-up in years; *ON*, osteonecrosis; *OA*, osteoarthritis

### Quality assessment (risk of bias and level of evidence (LoE))

Based on the OCEBM criteria [14], two studies were level 2b and 12 were level 3a (Table 1), with an overall grade B of recommendation assigned to the review [15]. The scores of all 14 studies ranged from 5 to 8, with an average of 7 +  − 0.9, indicating a low overall risk of bias. Twelve (86%) of the included studies were of good quality, while only two studies (14%) were of fair quality upon conversion to AHRQ standards. A summary of the qualitative assessment, according to the Newcastle–Ottawa scale, is shown in Table 2.

**Table 2:** Risk of bias was assessed using the Newcastle–Ottawa scale. A higher overall score indicates a lower risk of bias; a score of 5 or less (out of 9) corresponds to a high risk of bias

Study	Selection	Comparability	Outcome	Total score	AHRQ standards
1997, Xenakis	***	*	***	7	Good
1999, Ortiguera	***	**	**	7	Good
2006, Mont	***	**	***	8	Good
2008, Dastane	**	**	***	7	Fair
2009, Radl	***	*	***	7	Good
2014, Bergh	***	*	**	6	Good
2016, Ancelin	****	*	***	8	Good
2016, Liu	***	**	***	8	Good
2017, Singh	****	**	**	8	Good
2018, Osawa	***	*	***	7	Good
2019, Hart	***	**	**	7	Good
2020, Kumar	***	*	**	6	Good
2021, Sax	***	*	*	5	Fair
2022, Moharrami	***	*	**	6	Good

### Revisions

**Fig. 2:**
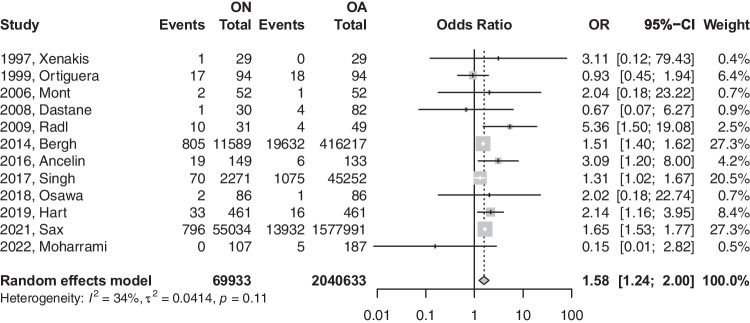
Forest plot comparison of the overall revision between ON and OA patients. CI, confidence interval; OR, odds ratio

**Fig. 3:**
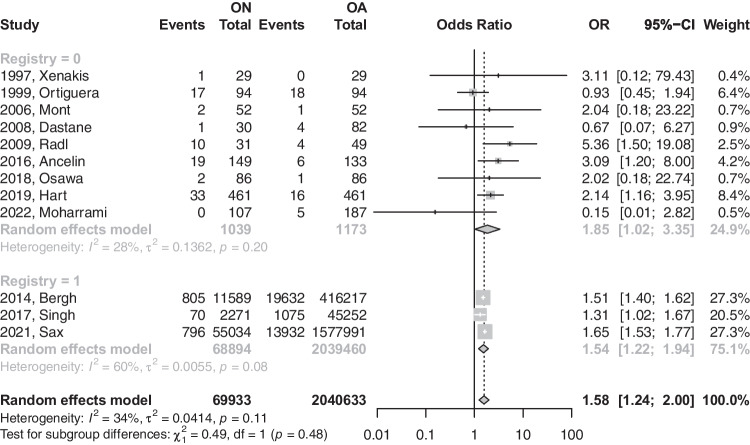
Forest plot comparison of the overall revision between ON and OA patients in registry versus non-registry studies. CI, confidence interval; OR, odds ratio

The primary analysis of the 12 eligible studies demonstrated that patients with (ON) were 1.58 times more likely to have a revision after THA (OR: 1.576; 95%CI: 1.24–2.00; *p*-value: 0.0015) [refer to Fig. 2]. Upon removing registry-based studies, a total of nine studies demonstrated that patients with ON are 1.84 times more likely to have a revision than their osteoarthritis (OA) counterparts after THA (OR: 1.847; 95%CI: 1.01–3.34; *p*-value: 0.0445) [refer to Fig. 3].

The earlier model had a heterogeneity value of 34.4%, which was insignificant (*p*-value = not significant (NS)), while the latter model had a heterogeneity value of 27.9% at a *p*-value of 0.1966. Further sub-analysis based on the modes of failures was performed to further explore the differences in revision reasons across both groups (Table 3).

**Table 3:** Comparison based on failure modes of revision surgeries

	ON	OA			
Mode of THA failure	Events/total	Events/total	OR or MD (95% CI)	Heterogenity (*I*^2^)	*P*-value
Aseptic loosening [Fig B]	247/12,622	4034/417,143	OR: 1.69; 95%CI: 0.59–4.83	69%	0.2723
PJI [Fig C]	996/70,059	19,835/2,040,528	OR: 1.459; 95%CI: 1.298–1.641	11%	** < 0.0001**
Instability [Fig D]	19/729	13/568	OR: 0.804; 95%CI: 0.043–14.92	*67%*	0.7794
Periprosthetic fracture [Fig E]	111/14,927	1288/462,429	OR: 2.137; 95%CI: 1.769–2.582	0%	** < 0.0001**

*OR*, odds ratio; *MD*, mean difference

### Dislocations

**Fig. 4:**
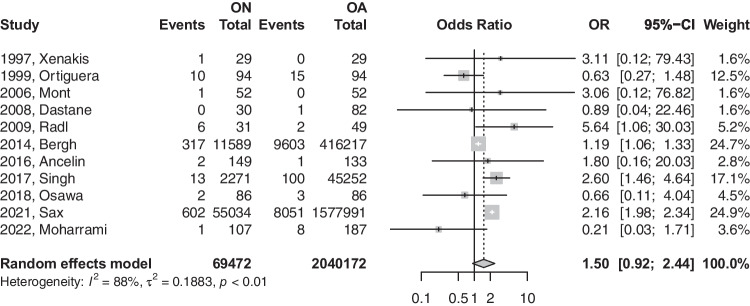
Forest plot comparison of the overall dislocation between ON and OA patients. CI, confidence interval; OR, odds ratio

**Fig. 5:**
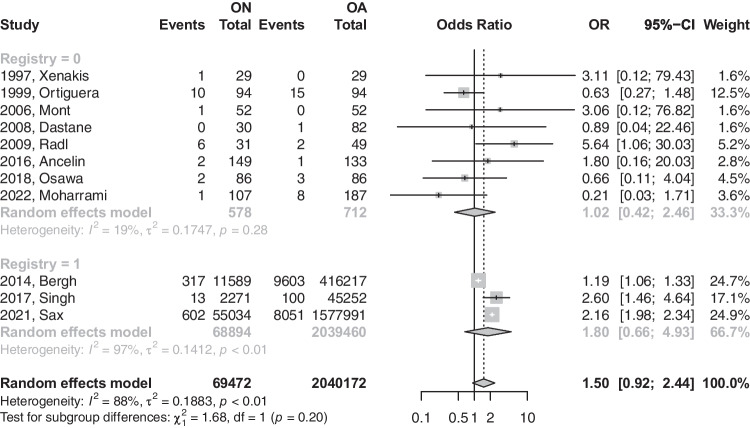
Forest plot comparison of dislocation between ON and OA patients in registry versus non-registry studies. CI, confidence interval; OR, odds ratio

The number of dislocations was reported by 11 studies for both ON and OA groups after THA. Patients with ON were 1.5 times more likely to experience a dislocation. However, that effect was statistically insignificant (OR: 1.5004; 95%CI: 0.92–2.43; *p*-value = 0.0916) [refer to Fig. 4]. This difference in risk was reduced to near equivalence with the removal of registry-based studies (OR: 1.02; 95%CI: 0.42–2.46; *p*-value: 0.9607) [refer to Fig. 5]. Upon the removal of registry-based studies, heterogeneity was reduced from 88.0 to 19.4%.

### HHS

**Fig. 6:**
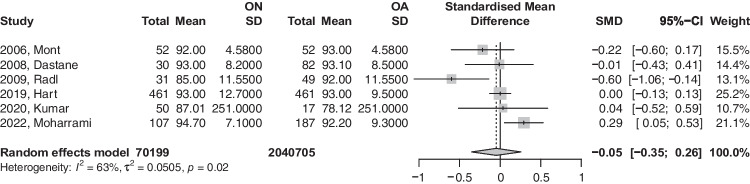
Forest plot comparison of functional Harris Hip Score between ON and OA patients. CI, confidence interval; SMD, standardized mean difference

**Fig. 7:**
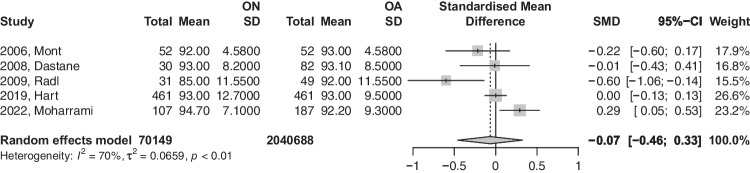
Sub-analysis of functional Harris hip score between ON and OA patients with normal distribution of effect size values. CI, confidence interval; SMD, standardized mean difference

A total of six studies had reported HHS. Our analysis, as demonstrated in Fig. 6, showed that patients with ON had slightly reduced HHP scores compared to the OA group, an effect that was statistically insignificant (SMD: − 0.0486; 95%CI: − 0.35–0.25; *p*-value = 0.6987). Based on the recommendation of Shi et al. (2020), the study titled (2020, Kumar) was removed as it violates normal distribution of effect size values. The analysis of this subgroup is provided in Fig. 7 and did show no deviance from the conclusions of the first model.

### Publication bias

**Fig. 8:**
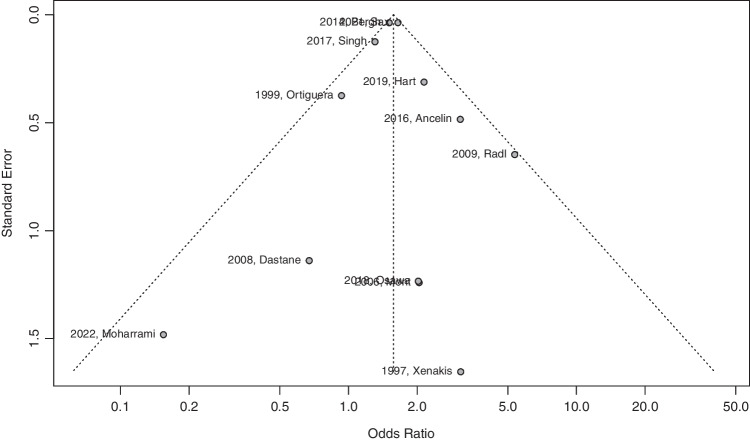
Egger’s test of symmetry displaying publication bias

Egger’s test of symmetry demonstrated that our set of 12 eligible studies displayed no publication bias (intercept: 0.027; 95%CI: − 0.85–0.91; *t*: 0.06; *p*-value: 0.95) [refer to Fig. 8].

## Discussion

The main findings of this review were that the revision rate, periprosthetic fracture, and periprosthetic joint infection were higher following THA for AVN than for primary OA. However, dislocation rates and functional outcomes, using HHS, were comparable.

### Revision

As demonstrated, patients with ON were statistically more likely to have revision procedures following their index procedure than THA for OA [16, 17]. The reasons for this are not clearly defined within the literature but can be theorized in relation to patients and surgical and implant factors.

The mean age of those in the ON cohort was lower than that performed for OA [17, 18]. This, in turn, supports the notion that patients undergoing THA for ON cycle their prostheses over a more extended period due both to their age and increased activity level, thus requiring revision at higher rates than the OA cohort [19–21].

Given the increased revision rate evident in this patient cohort, an emphasis should be placed on attentive follow-up of these patients so that complications can be recognized in a timely fashion.

The inclusion of registry data both adds impetus in relation to data volume but is also limited due to the nature of registry data [22]. Registry data defines revision as a one-time set end point and does not accommodate for decision-making surrounding whether a revision is warranted or not. Elderly patients with multiple co-morbidities may be candidates for revision on the basis of radiographs and clinical presentation; however, their medical status and age may preclude them from an appropriate revision operation [21, 22]. Similarly, patients in the ON cohort are younger and more active, and thus revision operations may be offered more readily in this cohort [18, 21].

### Dislocations

The cohort undergoing THA for ON demonstrated a statistically insignificant higher dislocation rate than OA patients. Anceilin et al. have postulated that patients undergoing ON have less soft tissue constraint in comparison to OA patients [21]. This could be due to a reduction in bone quality leading to changes in soft tissue quality surrounding the hip and thus de-tensioning of the soft tissue envelope. No quantitative analysis of this is available to substantiate this theory; however, other evidence may support this finding. Given the higher functional status of patients undergoing THA for ON, the ability to put themselves in the extremes of range of motion on a consistent basis may indeed confer a higher dislocation rate [20, 21, 23]. The increased rate of dislocation in the ON cohort will naturally feed into the overall revision rate and should be considered a contributory factor in this regard.

### Periprosthetic fracture

The rate of periprosthetic fracture (PF) was twice as high in the ON group compared to the OA cohort, reaching statistical significance (OR: 2.137; 95%CI: 1.769–2.582; *p*-value: < 0.0001). The work of Zhu et al. corroborates this finding, with THR performed for OA deemed a protective factor against PF [24]. Zhu et al. have theorized that the relative deconditioning of a patient undergoing THR for OA may lead to a lower activity level and, thus, reduced rate of PF in comparison to a higher-level activity patient undergoing THA [24]. Further, as reported by Al Saleem et al., the aberrant metaphyseal anatomy of the femur may result in canal obliteration in ON patients, predisposing patients to a higher rate of PPF [25].

### Periprosthetic joint infection

The rate of periprosthetic joint infection (PJI) was statistically higher in the ON group in comparison to the OA cohort (OR: 1.459; 95%CI: 1.298–1.641; *p*-value: < 0.0001). The work of Ren et al. supports this notion, demonstrating through their meta-analysis that ON is an independent risk factor for PJI in comparison to OA, which was deemed protective [26, 27]. The underlying etiology for the ON of the femoral head may lead to systemic immunosuppression, for example, chronic corticosteroid use or irradiation, and thus provide a more suitable environment for PJI to manifest postoperatively. An awareness of this increased clinical risk in ON patients should lead to heightened awareness of this potentially devasting complication in the postoperative period for the treating clinician.

### Functional outcome (HHS)

The HHS was not statistically different between both cohorts. The reasons for this were not clearly explained in the literature. One possible theory relates to the routine, standardized protocols utilized in the perioperative care of THA. Emphasis on prehabilitation, physiotherapy, and postoperative rehabilitation has meant that THA patients receive uniform postoperative care, despite the initial surgical indication [20, 23, 28].

Similarly, the technical challenge of performing THA for ON was not clearly explained in the literature, with multiple studies reporting grossly similar radiological parameters in postoperative X-rays. This finding, combined with routine postoperative protocols, supports the finding of similar functional outcomes in both cohorts of patients [29].

The huge sample size, long follow-up periods, high quality (low risk of bias) of the included studies, and the inclusion of all THR prosthesis designs (hybrid, cementless, and cemented) were all strength points that enhanced the external validity and generalizability of our results.

Although this review has many strengths, several limitations must be acknowledged. First, the ON cohort was analyzed regardless of the distinct underlying etiology, for example, steroid use or alcohol consumption [21, 22]. Second, subgroup analysis based on ON etiology would have eliminated the heterogeneity of this condition and the potential impact on the overall outcome of THA [30]. However, this was not possible due to limited studies and inconsistent reporting of ON etiology in the literature.

Considering that the implant type and surgical factors might influence the outcomes of THR [17, 18], another weakness was the inadequate reporting of such factors within some of the pooled studies. Cohort and retrospective studies were included, representing the highest available evidence level. Future work should comprise prospective studies in order to better control these confounders and evaluate this question in a more statistically robust manner.

## Conclusion

This study demonstrated a significantly higher revision rate, periprosthetic fractures, and PJI in patients with femoral head osteonecrosis following total hip replacement compared to patients with primary osteoarthritis. However, dislocation rates and HHS functional outcome measures were comparable. This finding should be applied in context due to potential confounding factors and the heterogeneous causes of ON.

### Supplementary Information

Below is the link to the electronic supplementary material.

Supplementary file1 (DOCX 13 KB)

## Data Availability

Not applicable as this is a review article. However, happy to provide access to any statistical data (coding) upon request.

## Materials availability

Not applicable as this is a review article. However, happy to provide access to any statistical data (coding) upon request.

## Code availability

Not applicable as this is a review article. However, happy to provide access to any statistical data (coding) upon request.
