# Supplementary material for: The outcomes of total hip replacement in osteonecrosis versus osteoarthritis: a systematic review and meta-analysis
Source: Int Orthop. 2023 Mar 11;47(12):3043–52. doi: 10.1007/s00264-023-05761-6 (PMC10673986; doi:10.1007/s00264-023-05761-6)
Supplement: Supplementary file 1 — Supplementary file1 (DOCX 13 KB) [file 264_2023_5761_MOESM1_ESM.docx]

**Supplementary results**

Total failures

Overall, patients with ON were 1.66 times more likely to experience revision failures (OR: 1.655; 95%CI: 1.0026 – 2.7347; p-value: 0.0490) [Refer to **Figure A**]. Upon the removal the registry-based studies, the OR increased to 2.00 times, however, became statistically insignificant (OR: 2.00; 95%CI: 0.90 – 4.44; p-value: 0.0788) [Refer to **Figure A_2**].

Aseptic Loosening

In terms of Aseptic Loosening, patients with ON were 1.69 times more likely to report Aseptic Loosening. Nonetheless, the difference is statistically insignificant (OR: 1.69; 95%CI: 0.59 – 4.83; p-value: 0.2723) [Refer to **Figure B**]. Upon the removal the registry-based studies, the OR increased to 1.72 times, however, became statistically insignificant (OR: 1.718; 95%CI: 0.44 – 6.58; p-value: 0.3620) [Refer to **Figure B_2**].

PJI

Moreover, patients with ON were 1.46 times more likely to present with failure due to PJIs (OR: 1.459; 95%CI: 1.298 – 1.641; p-value: <0.0001) [Refer to **Figure C**]. Upon the removal the registry-based studies, the OR increased to 2.12 times and retained its statistical significance (OR: 2.12; 95%CI: 1.096 – 4.094; p-value: 0.0317) [Refer to **Figure C_2**].

Instability

Only a handful of studies reported on instability. Patients with OA were more likely to present with failure of revision due to instability (OR: 0.804; 95%CI: 0.043 – 14.92; p-value: 0.7794) [Refer to **Figure D**].

Periprosthetic Fractures

Finally, patients with ON were 2.14 times more likely to present with periprosthetic fractures (OR: 2.137; 95%CI: 1.769 – 2.582; p-value: <0.0001) [Refer to **Figure E**]. Upon the removal the registry-based studies, the OR increased to 2.51 times while retaining its statistical significance (OR: 2.505; 95%CI: 1.202 – 5.221; p-value: 0.0222) [Refer to **Figure E_2**].

Heterogeneity

For Total Failures, Aseptic Loosening, and Instability, heterogeneity was significantly present and was at least above 60%. In contrast, heterogeneity for PJI and Periprosthetic fractures were insignificant ranging from 11% to 0%.
